# The dual orexin receptor antagonist TCS1102 does not affect reinstatement of nicotine-seeking

**DOI:** 10.1371/journal.pone.0173967

**Published:** 2017-03-15

**Authors:** Shaun Yon-Seng Khoo, Gavan P. McNally, Kelly J. Clemens

**Affiliations:** School of Psychology, University of New South Wales, Sydney, Australia; Radboud University Medical Centre, NETHERLANDS

## Abstract

The orexin/hypocretin system is important for appetitive motivation towards multiple drugs of abuse, including nicotine. Both OX_1_ and OX_2_ receptors individually have been shown to influence nicotine self-administration and reinstatement. Due to the increasing clinical use of dual orexin receptor antagonists in the treatment of disorders such as insomnia, we examined whether a dual orexin receptor antagonist may also be effective in reducing nicotine seeking. We tested the effect of intracerebroventricular (i.c.v.) administration of the potent and selective dual orexin receptor antagonist TCS1102 on orexin-A-induced food self-administration, nicotine self-administration and reinstatement of nicotine-seeking in rats. Our results show that 30 μg of TCS1102 i.c.v. abolishes orexin-A-induced increases in food self-administration but does not reduce nicotine self-administration. Neither i.c.v. 10 μg nor 30 μg of TCS1102 reduced compound reinstatement after short-term (15 days) self-administration nicotine, but 30 μg transiently reduced cue/nicotine compound reinstatement after chronic self-administration (29 days). These results indicate that TCS1102 has no substantial effect on motivation for nicotine seeking following chronic self-administration and no effect after shorter periods of intake. Orexin receptor antagonists may therefore have little clinical utility against nicotine addiction.

## Introduction

Tobacco smoking is the leading cause of preventable disease in North America and Western Europe, where it accounts for one in five deaths [1–3]. Existing therapies such as partial nicotinic receptor agonists are somewhat effective, yet relapse rates remain high [4]. This suggests that further research into potential therapeutic targets is warranted and necessary. The orexin/hypocretin system may be a potential therapeutic target because it is activated by acute nicotine administration [5]. It has also been widely implicated in the appetitive motivational properties for several drugs of abuse, including alcohol, cocaine, and opioids, as well as non-drug reinforcers such as high-fat food [6–8].

Orexin/hypocretin neurons originate exclusively from the lateral, dorsomedial and perifornical areas of the hypothalamus [9, 10] and project widely throughout the neuraxis, innervating key reward regions such as the ventral tegmental area and nucleus accumbens [11, 12]. In addition to expressing the hypocretin gene (*Hcrt*) that encodes the precursor for both orexin-A and orexin-B, they also contain co-transmitters such as glutamate [13], dynorphin [14] and neurotensin [15]. Notably, orexin/hypocretin neurons in the lateral and perifornical area of the hypothalamus have been shown to be activated by acute nicotine [5, 16, 17], suggesting a potential role for orexins in nicotine dependence.

This role is supported by antagonist studies showing that orexins have multiple functions in appetitive motivation for nicotine that are differentially mediated by its two receptors, OX_1_ and OX_2_. The orexin system’s two G-protein coupled receptors, OX_1_ and OX_2_, have both been implicated in operant nicotine seeking (reinstatement) and taking (self-administration). Systemic administration of the selective OX_1_ receptor antagonist SB-334867 has been shown to reduce operant intravenous self-administration of nicotine under both fixed (FR5) and progressive ratio schedules in Wistar rats [18]. Further, nicotine alters expression of *Hcrt1* mRNA that encodes for the OX_1_ receptor [19]. Studies in mice have found similar effects with SB-334867 reducing signs of withdrawal and cue-induced reinstatement of operant intravenous self-administration [20, 21]. The consistency of results across strains and species implicates the OX_1_ receptor in both nicotine seeking and taking behaviours.

Studies targeting the OX_2_ receptor suggest a similar but more limited role in appetitive motivation for nicotine. A selective OX_2_ receptor antagonist decreases cue-induced reinstatement of operant nicotine seeking in Long Evans rats, but not nicotine-primed reinstatement or progressive ratio self-administration of nicotine [22]. However, in mice, the OX_2_ receptor antagonist TCSOX229, has no effect on cue-induced reinstatement [21]. Although there are few published studies, the lack of consistent findings suggests the OX_2_ receptor may have a more limited and selective role in nicotine seeking behaviour than the OX_1_ receptor.

Targeting both receptors may also be an effective strategy for reducing nicotine seeking. For example, almorexant, a dual orexin receptor antagonist, has been shown to be effective in reducing conditioned place preference of stimulant drugs like amphetamine and cocaine [23]. Almorexant also reduces operant alcohol self-administration in rats [24]. Moreover, TCS1102 is a potent and selective dual orexin receptor antagonist and member of the same drug class as the recently approved suvorexant and has been shown to reduce nicotine reinstatement without affecting food reinstatement [25, 26]. A dual orexin receptor antagonist may therefore be an effective method of reducing nicotine seeking behaviour.

The aim of the present study was to examine the role of dual orexin antagonism in regulating nicotine seeking in rats. Since the neurobiology of orexins and nicotine can change with chronic exposure to drugs [27, 28] we tested rats after short-term or chronic periods of access. We demonstrated the efficacy of TCS1102 against orexin-A-induced food self-administration and then tested the effects of TCS1102, on nicotine self-administration and reinstatement in rats given chronic and short-term access to nicotine.

## Materials and methods

### Subjects

Experimentally naive male Sprague-Dawley rats (n = 48) were obtained from the Animal Resources Centre (Perth, WA, Australia) at 6 weeks of age weighing 240-280g. Rats for experiment 2 (n = 16) were housed in individually ventilated cages (309 mm wide, 617 mm long, 284 mm high) with a mezzanine floor under a 12 h: 12 h light/dark cycle (lights on at 07:00). Rats for all other experiments were housed in groups of four in plastic tubs (24 cm high × 64 cm long × 40 cm wide) with wire tops, corncob bedding and environmental enrichment (aspen blocks for chewing, plastic tunnels and cardboard) under a reverse 12 h: 12h light/dark cycle (lights off at 06:00) with *ad libitum* food and water prior to behavioural testing. Rats were given 1 week to acclimate to the colony room and then handled daily for 5 days before undergoing surgery and behavioural testing. 2 days prior to commencement of self-administration training, rats were placed on a mild food restriction schedule (20-22g/rat/day).

All procedures were approved by the University of New South Wales Animal Care and Ethics Committee and conducted in accordance with the National Health and Medical Research Council’s Australian Code for the Care and Use of Animals for Scientific Purposes (8^th^ edition).

### Drugs

Nicotine was obtained from Sigma-Aldrich (Castle Hill, NSW, Australia), dissolved in 0.9% sterile saline and pHed to 7.4 with sodium hydroxide. All concentrations are expressed as the base.

TCS 1102 (N-(1,1'-Biphenyl)-2-yl-1-(2-((1-methyl-1H-benzimidazol-2-yl)thio)acetyl-2-pyrrolidinedicarboxamide; Batch No: 2A/155485; MW: 475.09) and orexin-A (OX-A; Batch No: 13D/179299; MW: 3561.12) were obtained from Tocris Bioscience (Bristol, United Kingdom). The vehicle solutions were (2-hydroxypropyl)-β-cyclodextrin obtained from Sigma-Aldrich (Castle Hill, NSW, Australia; Lot #112K4614) diluted with saline to a final concentration of 20% (2-hydroxypropyl)-β-cyclodextrin, 0.5% saline and 20% D-α-tocopherol polyethylene glycol 1000 succinate, (Vitamin E-TPGS; Sigma-Aldrich, Castle Hill, NSW, Australia; Lot #BCBM7443V) in 0.9% saline. The Vitamin E-TPGS solution allowed a higher dose to be delivered, but the β-cyclodextrin vehicle was less viscous and easier to prepare. TCS1102 was stored at room temperature according to the manufacturer’s instructions and solutions were freshly prepared for each test. Orexin-A was dissolved at a concentration of 0.5 μg/μL in 0.9% saline and stored at -20C until use.

### Surgery

A single surgery was performed to implant both the chronically indwelling jugular vein catheters as previously described [29] and a single guide cannula targeting the lateral ventricle, or just the single guide cannula as required. Briefly, rats were deeply anaesthetized using 3% isoflurane and given a pre-emptive analgesic injection of carprofen (5 mg/kg, s.c.). The catheter was implanted such that it passed from a back mount to the right jugular vein in the neck and then it was secured and flushed with 0.2 mL of 100 mg/mL cephazolin. Rats remained under anaesthesia while being transferred to a stereotaxic frame (David Kopf Instruments, Tujunga, CA, U.S.A.). Local anaesthetic (bupivacaine) was applied to the head and a guide cannula (26 ga; PlasticsOne, Roanoke, VA, U.S.A.) was implanted targeting the lateral ventricles using the following coordinates (from Bregma): -0.8 mm anteroposterior (A/P), +1.5 mm mediolateral (M/L) and -4 mm dorsoventral (D/V). Rats were given 2 days of post-operative carprofen (5 mg/kg, s.c.) and 7 days recovery, during which time they were monitored and weighed daily to ensure they did not lose more than 20% of preoperative bodyweight, before behavioural experiments. The catheter was flushed daily with heparinised (150 IU/mL) cephazolin (0.2 mL, 100 mg/mL) until the end of behavioural testing.

### Experiment 1: Orexin-A-induced food self-administration

To simulate free-feeding, rats (n = 16) were trained for 10 days in daily 2 h operant self-administration sessions for 45 mg grain pellets (#F0165, BioServ, Flemington, NJ, USA). Training took place in 8 identical operant chambers (Med Associates, St Albans, VT, U.S.A.). Each chamber had a fan, a houselight to provide gentle illumination on one side and a magazine on the opposite side. Magazine entries produced delivery of food pellets on a variable interval 20 s schedule. Food pellets were delivered to the magazine. For 4 days prior to their first test day, rats were habituated to the microinjection procedure by having their obturators removed and replaced and gently restrained as they would be for microinjections before their operant session. They then received microinjections of 5 μL 0.9% saline, OX-A/Vitamin E-TPGS and OX-A/TCS1102 on separate days using a within-subjects design. At least 1 day of normal food self-administration was given between test days to ensure test effects had washed out. All microinjections were in volumes of 5 μL and delivered over 2 min, with injectors left in place for an additional 30 s to allow the solution to circulate. On the double microinjection days, rats received 2.5 μg of OX-A in 5 μL and were returned to their home-cage for 5 min. They then received the 30 μg of TCS1102 in 20% Vitamin E-TPGS vehicle or vehicle alone and were returned to their home-cage for another 10 min before being placed in the operant chamber for behavioural testing.

### Experiment 2: Self-administration

#### Acquisition

Rats for Experiments 2–4 were trained for operant self-administration of nicotine in 8 identical operant chambers (Med Associates, St Albans, VT, U.S.A.). These chambers were identical to those used in Experiment 1, except they also had two nosepoke holes. For half of the chambers, the left nosepoke was active and for the other half the right nosepoke was active. Additionally, these chambers was equipped with four evenly spaced photobeam sensors to monitor locomotor activity during operant sessions.

Rats received 2 days of 1 h habituation sessions in the self-administration chambers. During these sessions the nosepokes were covered, the houselight was on, and rats were not connected to the infusion line.

Rats then received daily 1 h sessions of nicotine self-administration. The nosepokes were uncovered and responses on the active nosepoke resulted in a 3 s infusion of nicotine (30 μg/kg/infusion), illumination of the nosepoke cue light (3 s) and the houselight was switched off during a 20 s time-out period. Responses made during the timeout period, or on the inactive nosepoke, were counted but had no programmed consequences.

**Fig 1:**
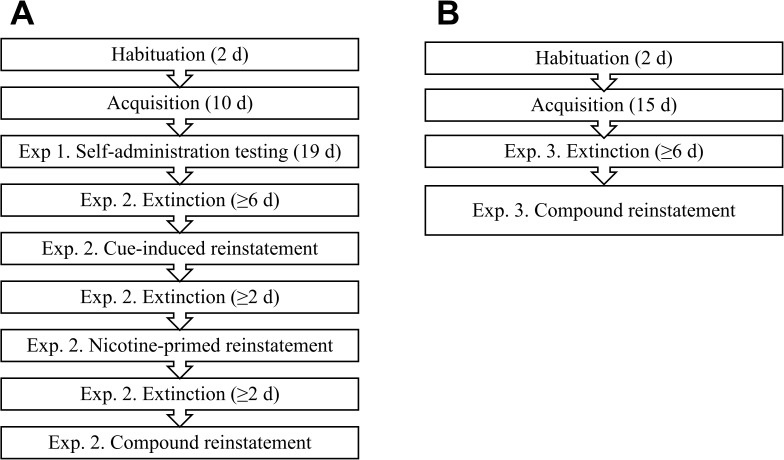
Experimental procedures. (A) In Experiments 2 and 3, rats were first trained for nicotine self-administration. They were then tested for self-administration before undergoing extinction and tests for cue-induced, nicotine-primed and cue/nicotine compound reinstatement. (B) In Experiment 4, rats were first trained for nicotine self-administration for 15 days. They then underwent extinction and testing for cue/nicotine compound reinstatement.

During this acquisition phase, training was conducted 5 days per week and rats were considered to have acquired if they achieved at least 6 infusions of nicotine during the 60 min session and at least 2:1 discrimination for the active nosepoke. After the commencement of drug testing, all sessions took place on consecutive days. A summary of the procedure for Experiments 1 and 2 is presented in Fig 1A.

#### Self-administration testing

Once rats had experienced 10 acquisition sessions and were at stable responding (<30% variation in the number of infusions earned per day), they were given a series of microinjections to test the effects of TCS 1102 on nicotine self-administration. Procedures for habituation to microinjections were as described for Experiment 1.

They then received microinjections of vehicle (20% (2-hydroxypropyl)-β-cyclodextrin, 0.5% saline), 1μg, 3μg or 10μg TCS 1102 via a 33 ga injector which projected 1 mm beyond the guide cannula. TCS1102 was administered 10 min before the operant session, using a within-subjects design. 2 days of normal self-administration elapsed between test days to allow for washout and a return to baseline responding. The 10 μg TCS 1102 test was then repeated using a 20% Vitamin E-TPGS/0.9% saline vehicle to ensure that there was no difference between vehicle solutions.

### Experiment 3: Extinction and reinstatement after chronic access

Following the completion of Experiment 2, rats were placed on normal self-administration for 4 days, to ensure that they had returned to stable baseline responding. At this point they had received a total of 29 self-administration days and extinction training commenced.

During extinction sessions, the houselight and fan were on for the 1 h session but nosepokes had no consequences. Rats received at least 6 daily extinction sessions until they met extinction criteria which was active nosepoke responding of ≤30% of responding on the last day of self-administration for 2 consecutive days. Once extinction criteria was met they were tested for reinstatement. For each reinstatement test, rats were randomly assigned (between-subjects) to receive a microinjection of either Vitamin E-TPGS vehicle or 10 μg TCS 1102 10 min prior to the session.

Cue-induced reinstatement was precipitated by reinstating the response-contingent availability of the visual cues. The program was identical to the program used in training except that if a rat made no active nosepokes within the first 10 min a non-contingent cue presentation was delivered. Although rats were attached to the tethers, they did not receive infusions.

Rats then underwent a second phase of re-extinction until they met the extinction criteria again, and then were tested for nicotine-primed reinstatement. Nicotine primed reinstatement was precipitated by an injection of nicotine immediately before being placed in the chamber (0.3 mg/kg, 1 mL/kg, s.c.) and carried out under extinction conditions (houselight on, but all nosepokes were inactive). This procedure was then repeated for cue/nicotine compound reinstatement which involved a session identical to cue-induced reinstatement with a priming injection of nicotine (0.3 mg/kg, 1 mL/kg, s.c.) immediately before the session.

### Experiment 4: Compound reinstatement after short-term access

To examine whether the TCS1102 would affect reinstatement after short-term access, a separate cohort of rats (n = 16) underwent surgery, 15 days of acquisition and extinction in a manner identical to the rats in Experiments 2 and 3. Testing for cue/nicotine compound reinstatement procedures were also identical, except rats were tested following microinjection of vehicle, 10 μg or 30 μg TCS1102 using a within-subjects design. As for Experiment 3, there were at least 2 re-extinction sessions between reinstatement tests to allow rats to meet extinction criteria, as shown in Fig 1B.

### Histology

Once rats had completed all experiments, they were euthanized using a 0.2 mL IV infusion of 325 mg/mL sodium pentoparbitone via their catheters, therefore simultaneously verifying catheter patency. Rats in Experiment 1 who did not have jugular vein catheters were given an i.p. injection of sodium pentobarbitone combined with the local anaesthetic bupivacaine to reduce discomfort caused by pentobarbitone’s high pH [30].

To check the accuracy of the microinjections, cresyl violet was injected via guide cannulae in the same way as previous microinjections. The brain was then dissected out and coronal sections were cut using a razor blade. If ink was present in the ventricles, the microinjections were considered accurate, but if ink was absent from the ventricles, or was concentrated in the parenchyma, the rat was excluded.

The injection site of one animal, whose guide cannula became blocked before the cue/nicotine-primed reinstatement test, was validated by cryosectioning and staining to check the cannula track entered the ventricles.

### Data analysis

Statistical analysis was performed using SPSS 23.0 (IBM, New York, USA). Repeated measures ANOVA was used for Experiments 1, 2 and 4 and split-plot or mixed-design ANOVA was used for Experiment 3. For factors with more than two levels, Helmert contrasts were performed within the ANOVA because they are a coherent and statistically powerful type of orthogonal contrast that compares the control condition to all other conditions and then compares between the treatment conditions [31]. Data are presented as means ± SEM. Alpha was set at 0.05. All of the raw data presented in this manuscript is available on Figshare (doi: 10.6084/m9.figshare.3839646).

## Results

### Experiment 1: The dual orexin receptor antagonist TCS1102 abolishes orexin-A-induced food self-administration

**Fig 2:**
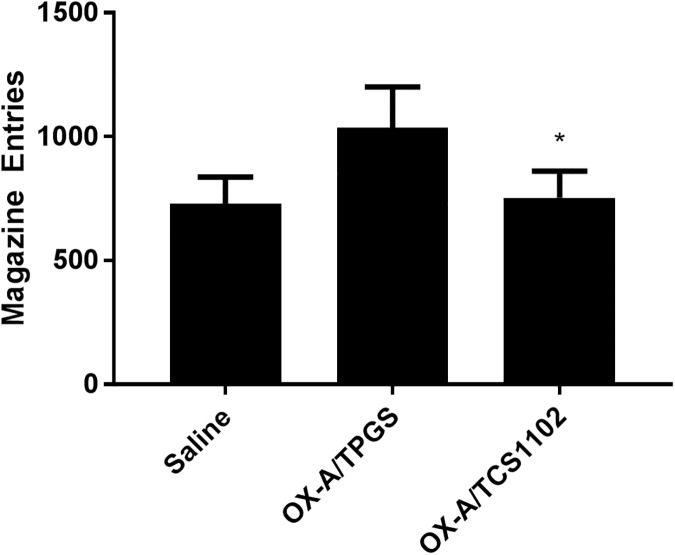
TCS1102 abolishes orexin-A-induced food self-administration. The number of magazine entries following administration of 2.5 μg OX-A and 30 μg of TCS1102 (OX-A/TCS1102) was significantly reduced compared to the 2.5 μg OX-A and 20% Vitamin E-TPGS (OX-A/TPGS) condition (n = 15). * *P* < 0.05 compared to OX-A/TPGS.

After 10 days of food self-administration rats made a mean of 582 ± 20 magazine entries, resulting in 57 ± 1.07 pellets delivered or 2.5 g of food. As shown in Fig 2, there was a significant main effect of test (F_(2,28)_ = 4.85, *p =* 0.016). Helmert contrasts showed that there was no difference between the saline test and 2 OX-A test days (F_(1,14)_ = 2.62, *p =* 0.127), however there was a significant difference in the number of magazine entries on the OX-A/Vitamin E-TPGS and OX-A/TCS1102 test days (F_(1,14)_ = 7.92, *p =* 0.014). These results demonstrate that TCS1102 is effective in reducing orexin-A-induced food self-administration.

### Experiment 2: The dual orexin receptor antagonist TCS1102 did not affect nicotine self-administration

After 10 days of self-administration, 13 rats met inclusion criteria. They made a mean of 21.9 ± 2.5 active nosepokes and 7.85 ± 1.68 inactive nosepokes, corresponding to 15.5 ± 1.44 infusions of 30 μg/kg nicotine during the operant session. There was a significant increase in active nosepoke responding over the course of training (F_(9,108)_ = 3.41, *p =* 0.001) but not for the inactive nosepoke (F_(4.5,108)_ = 2.1, *p =* 0.086) indicating that rats rapidly increased responding on the active nose-poke across training session.

**Fig 3:**
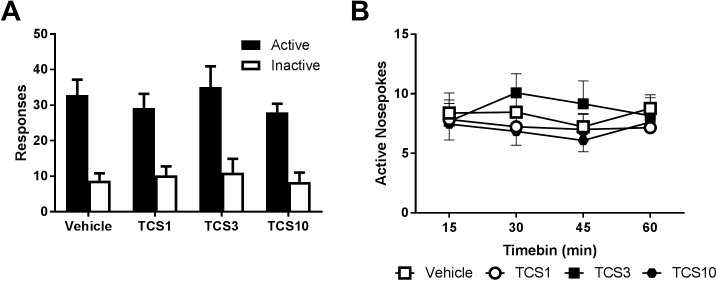
TCS1102 has no effect on nicotine self-administration. (A) TCS1102 had no effect on 1 h operant intravenous self-administration of nicotine in rats (n = 13) following intracerebroventricular microinjections of 1, 3 or 10 μg TCS1102. (B) Active nosepoke responding over the course of the session was also not altered.

Across self-administration, there was no effect of TCS1102 across the doses tested on responding for nicotine either overall (Fig 3A) or on the timecourse of responding during the session (Fig 3B).

There was also no effect on locomotor activity during the operant session, either overall or over the course of the session (Table 1). This result was then replicated (S1 Fig) using 10 μg TCS1102 and a 20% Vitamin E-TPGS/0.9% saline vehicle to facilitate the use of a higher dose of TCS1102 in subsequent experiments. The results of Experiment 2 show no effect of dual orexin receptor antagonism on nicotine self-administration.

**Table 1:** TCS1102 has no effect on locomotor activity during nicotine self-administration.

Timebin (min)	Vehicle	TCS1	TCS3	TCS10
15	6.2 ± 2.2	9.9 ± 3	4.8 ± 1.3	7.5 ± 2
30	18.7 ± 3.2	18.9 ± 2.7	17.6 ± 3.3	15.7 ± 3.8
45	6.2 ± 1.1	8.1 ± 1.5	5.9 ± 1.2	7 ± 1.1
60	15.1 ± 1.9	18.2 ± 2	14.8 ± 2.3	19.9 ± 2.8
Total	508.2 ± 41	527.8 ± 42.2	495.8 ± 40.4	484.2 ± 45.1

There was no effect of TCS1102 on the number of photobeam breaks recorded in the operant chamber during nicotine self-administration. Data are means ± SEM

### Experiment 3: TCS1102 did not affect cue-induced or nicotine-primed reinstatement

**Fig 4:**
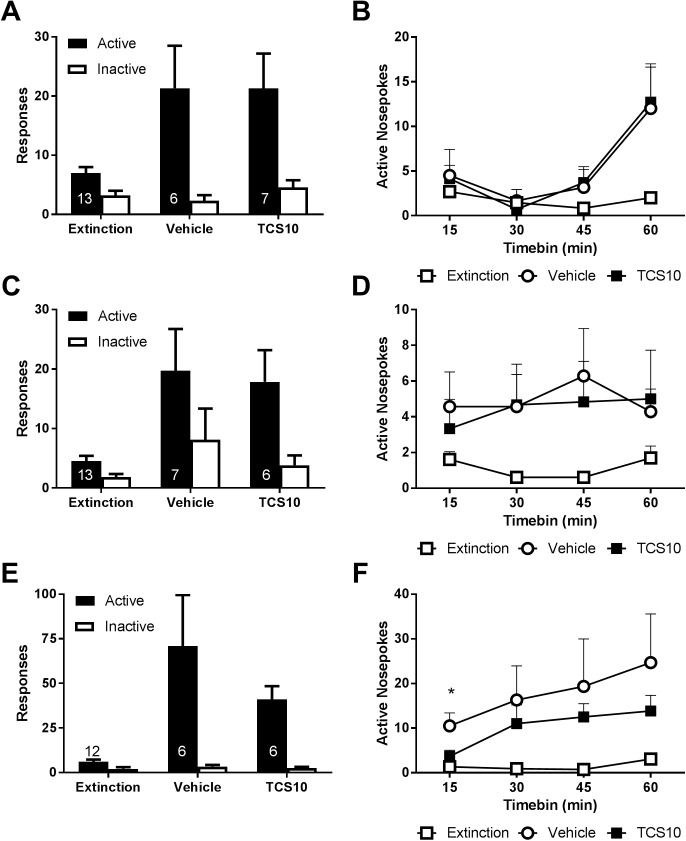
TCS1102 has only a transient effect on cue/nicotine compound reinstatement. (A) There was no effect on the magnitude of cue-induced reinstatement by centrally administered 10 μg of TCS1102. (B) There was also no effect on the timecourse of cue-induced reinstatement. (C) Similarly there was no effect of centrally administered 10 μg TCS1102 on overall responding or (D) the timecourse of active nosepokes during nicotine-primed reinstatement. (E) Although there was not a statistically significant reduction on overall responding during cue/nicotine compound reinstatement, there was a (F) small, transient reduction in active nosepokes during the first 15 minutes. * p < 0.05 for vehicle vs TCS10. Numbers on the figure indicate the allocation to each condition from a total of 12–13 rats per test. Data are means ± SEM.

As shown in Fig 4A, rats showed a robust cue-induced reinstatement of responding on the active nosepoke (session by nosepoke interaction (F_(1,11)_ = 9.72, *p =* 0.0098)). However, this was not influenced by treatment (Fig 4B).

Across nicotine-primed reinstatement, there was once again a robust reinstatement of responding as revealed by a main effect of session (F_(1,11)_ = 13.5, *p =* 0.0037), and a strong trend for session x nosepoke interaction (F_(1,11)_ = 3.45, *p =* 0.09). There was no effect of TCS1102 on the total number of active nosepokes (Fig 4C), nor was the time course of active nosepokes altered over the course of the session (Fig 4D).

Prior to the combined reinstatement test, one rat was excluded due to a blocked guide cannula. Again, rats showed robust reinstatement of active nosepokes during compound reinstatement as shown by a significant session x nosepoke interaction (F_(1,10)_ = 11.0, *p =* 0.008). Although there was no significant effect on overall responding during the session (F_(1,10)_ = 0.33, *p =* 0.58, Fig 4E), further examination of the data revealed a significant difference in responding across the first 15 minutes compared to the rest of the session (F_(1,10)_ = 4.99, *p =* 0.049). Post-hoc tests show that there was a significant difference in active nosepokes between vehicle and TCS1102 groups during the first 15 minutes of the compound reinstatement session (*p =* 0.039). The results of Experiment 3 show that while there was no effect on cue-induced or nicotine-primed reinstatement, there was a transient effect during the first 15 minutes of the compound reinstatement session.

### Experiment 4: TCS1102 does not affect compound reinstatement after short-term access

To examine whether this transient effect could be observed under short-term access to nicotine, we tested a separate cohort of rats (n = 14 met inclusion criteria). After 15 days of nicotine self-administration, rats made a 26.4 ± 4.17 active nosepokes, 5.64 ± 1.43 inactive nosepokes and received 17.6 ± 2.57 infusions of nicotine. Over the course of acquisition there was a significant day x nosepoke interaction (F_(14,182)_ = 7.76, *P* < 0.001) with the number of responses increasing over time (F_(14,182)_ = 9.97, *P* < 0.001) and more for the active nosepoke than inactive (F_(1,13)_ = 65.1, *P* < 0.001).

A repeated measures ANOVA with a Helmert contrast comparing the final extinction session to the reinstatement sessions revealed that there was a significant interaction with the nosepoke (F_(1,13)_ = 10.6, *p =* 0.006), indicating a robust reinstatement of active nosepoke responding.

**Fig 5:**
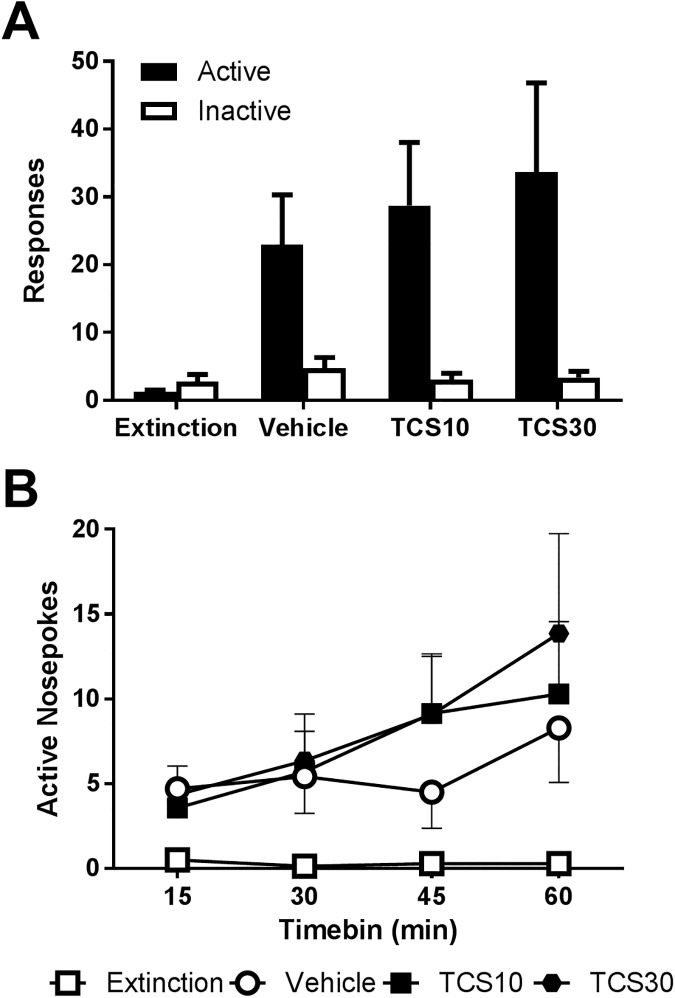
TCS1102 has no effect on cue/nicotine compound reinstatement after short-term self-administration. (A) In a separate cohort of rats (n = 14) trained for 15 days, cue/nicotine primed reinstatement was not affected by central administration of 10 or 30 μg of TCS1102. (B) TCS1102 also had no effect on active nosepokes over the course of the session. Data are means ± SEM.

As shown in Fig 5A, there was no significant difference between vehicle and TCS1102 treatment conditions in active nosepoke responding overall (F_(1,13)_ = 0.57, *p =* 0.46) or during the course of the session (Fig 5B). The results of Experiment 4 indicate that TCS1102 had no effect on compound reinstatement after 15 days of nicotine self-administration.

## Discussion

The results of the present study show that the dual orexin antagonist TCS1102 is effective in blocking orexin-A-induced food self-administration but has no substantial effect on nicotine-seeking. TCS1102 had no effect on nicotine self-administration, cue-induced and nicotine-primed reinstatement and but had a small, transient reduction in nicotine-seeking following cue/nicotine compound reinstatement in rats given chronic, but not short-term access to nicotine self-administration.

The finding that TCS1102 affected nicotine-seeking in rats given chronic but not short-term periods of access to nicotine is consistent with the literature. Several studies have shown that the degree of addiction-like behaviours that an animal exhibits, such as seeking despite adverse consequences, increases with chronic access [32, 33]. For nicotine, it has been shown that rats given chronic access will engage in longer bouts of operant responding to earn more infusions [34]. These behavioural changes are accompanied by neurobiological changes as well, such as an upregulation of nicotinic ACh receptors [28]. The orexin system also becomes more excitable as it is exposed to stimulant drugs [35, 36]. These neuroplastic changes may alter the degree to which the orexin system is involved in nicotine-seeking, which would explain why we found an effect on nicotine-seeking after chronic access, but not short-term access.

Rats given chronic access may also be more highly motivated for nicotine. Previous studies have shown that orexin antagonists decrease motivation in progressive ratio tests for cocaine [8], alcohol [37], and nicotine [18]. Orexin antagonists have also previously been shown to selectively affect high responders [38]. In the present study, rats escalated their responding during reinstatement sessions or had stable responding during the session, rather than making most of their responses at the beginning of the session as is typical for cocaine reinstatement [39]. This escalating pattern was evident in cue-induced and compound reinstatement in Experiment 3 and Experiment 4 and may reflect conditioned reinforcement because of the presence of cues but not nicotine [40]. It is therefore possible that rats in the present study had generally low levels of motivation for nicotine itself and that future studies using more highly motivated animals could see effects of orexin antagonism.

Intriguingly, our results do not suggest that cues alone are sufficient to provoke orexinergic regulation of nicotine-seeking. In contrast with previous studies, which showed effects of OX_2_ antagonism on cue-induced but not nicotine-primed reinstatement [22], we found that TCS1102 affected neither cue-induced nor nicotine-primed reinstatement and only transiently reduced cue/prime compound reinstatement. This is surprising, given that one of the functions of chronic access is to make rats susceptible to cue-induced reinstatement [34]. However, the rats in previous studies had also undergone extensive prior training prior to cue-induced reinstatement including a sequence of steps from FR1 food pre-training, FR1 to FR2 and FR5 nicotine responding and then a period of progressive ratio training before being tested for reinstatement [22]. This involved a minimum of 16 days on FR training for nicotine before extinction, not including progressive ratio training which continued until responding was stable. Rats in the present study had only undergone training on FR1 nicotine self-administration and testing of self-administration behaviour. Moreover, rats in the present study underwent surgery before any operant training took place and were group-housed, while previous studies have performed surgery after the food pre-training phase and singly-housed their rats [22]. Rats in the present study also received i.c.v. microinjections, which require the animal to undergo more handling than oral delivery, but do not expose the drug to first-pass hepatic metabolism or require it to cross the blood brain barrier. The different training histories and testing protocls between the two studies make it difficult to draw strong conclusions because rats that have been trained under multiple schedules or experience a different level of stress due to handling or housing conditions may assign a different level of salience and motivational strength to the nicotine and its associated cues.

Different training histories and testing protocols may also explain why there was no effect on nicotine self-administration. All of the previously discussed operant nicotine intravenous self-administration studies in rats have used an extensive food pre-training protocol and an FR5 schedule, although they typically do not state how many sessions of food pre-training are used [18, 19, 22]. However, in our protocol rats never associated their operant responses with obtaining food and only ever associated the context of the operant chamber with nicotine rewards. It has now been shown that rats prefer sweet rewards to nicotine, even after a chronic period of access [41], consistent with findings comparing preferences for natural reinforcers to drug reinforcers like cocaine and heroin [42]. By pre-training rats with food and using an FR5 schedule, it is possible that previous studies have enhanced the salience which animals assign to the context and cues in the operant chamber. Moreover, when nicotine is used to precipitate reinstatement of operant responding for food-associated cues TCS1102 can attenuate reinstatement [26]. These effects might persist even though these studies do not necessarily provide more days of self-administration than were used in the present study. In one study, pair-housed rats received 7–14 days of nicotine self-administration before tests involving microinjections of SB-334867 [18], while another gave singly-housed rats 40–66 days of nicotine self-administration before beginning tests of systemic SB-334867 (i.p.) and orally administered almorexant [19]. The differences in these training histories and testing protocols strongly suggest a need for further studies to examine the role of food pre-training, length of nicotine self-administration training, and testing protocols on orexinergic modulation of nicotine self-administration.

Pharmacological differences may also explain why the present study did not show effects of orexin antagonism on nicotine self-administration, but this is unlikely. We chose TCS1102 because of its high potency and selectivity for the orexin receptors. As previously reported, TCS1102 reduces orexin-A-induced Ca^2+^ at 17 and 4nM concentrations for the OX_1_ and OX_2_ receptors respectively [25]. TCS1102 is highly selective for the orexin receptors, with only three hits in μM range reported on an MDS Pharma screen for a monoamine transporter, thromboxane A_2_ and thyrotropin releasing hormone. While its half-life is quite short, at approximately 20 min, it is comparable to the 24 min half-life of SB-334867 [43] and behavioural effects for TCS1102 have been reported following systemic doses as low as 15 mg/kg and these persist throughout a 180 min locomotor session [25]. Our doses of 10–30 μg administered i.c.v. should avoid hepatic metabolism and produce brain-concentrations equivalent to those achieved by effective systemic doses. Indeed, we have shown that i.c.v. TCS1102 abolishes OX-A-induced food self-administration, demonstrating that TCS1102 is acting in the brain to block orexin signalling. We administered 2.5 μg of orexin-A to increase food self-administration behaviour and this was blocked by 30 μg TCS1102. Concentrations of exogenously administered orexin-A are likely to be much higher than concentrations of endogenous orexin-A, which are usually reported in pg/mL [44–46]. If TCS1102 is acting as a competitive antagonist, this suggests that it is able to displace orexin-A even at elevated concentrations. However, it is also possible that the low endogenous concentrations of orexin-A indicate that only very small orexin signals are required for natural behaviour. Therefore, while this finding demonstrates that TCS1102 works as an orexin antagonist, it does not provide direct evidence that i.c.v. TCS1102 is able to reduce natural orexin-dependent behaviours. However, overall it seems unlikely that our lack of observed effects on self-administration and other types of reinstatement are due to off-target non-orexinergic effects, potency, half-life or vehicle solution.

However, it is possible that the differential pharmacokinetics of TCS1102 and other orexin receptor antagonists are involved. There are currently no published reports on the antagonist mechanism of TCS1102. In contrast, it has been shown that SB-334867 is a non-competitive antagonist [47] and almorexant may also be a non-competitive antagonist [47, 48]. However, it is unclear whether this non-competitive binding is due to irreversible antagonism or allosteric modulation [48]. It is possible that the differences in results between studies have been affected by the different mechanisms by which these antagonists act, especially if non-competitive antagonism may mean that commonly used orexin antagonists like almorexant and SB-334867 are biasing the intracellular signalling cascades triggered by the endogenous orexin peptides differently to TCS1102. This remains an important issue to be explored.

## Conclusions

We have demonstrated that the dual orexin receptor antagonist, TCS1102, blocks orexin-A-induced food self-administration but has only a small, transient effect on cue/nicotine compound reinstatement after chronic access to nicotine. These findings suggest that the orexin system may change its role in regulating appetitive motivation for a reinforcer over time. Although we did not find evidence that the orexin system regulates FR1 nicotine self-administration, or cue or nicotine-primed reinstatement alone, this may be due to subtle differences in TCS1102’s pharmacology or the levels of motivation and salience assigned to nicotine-associated cues. Our results do not support targeting the orexin system for addiction pharmacotherapy, but there may still be a subtle role for the orexin system in appetitive motivation and reward that is worthy of further investigation.

## Supporting information

S1 Fig (PDF)
