# Supplementary material for: The dual orexin receptor antagonist TCS1102 does not affect reinstatement of nicotine-seeking
Source: PLoS One. 2017 Mar 15;12(3):e0173967. doi: 10.1371/journal.pone.0173967 (PMC5351999; doi:10.1371/journal.pone.0173967)
Supplement: S1 Fig — (PDF) [file pone.0173967.s001.pdf]

Supporting Information

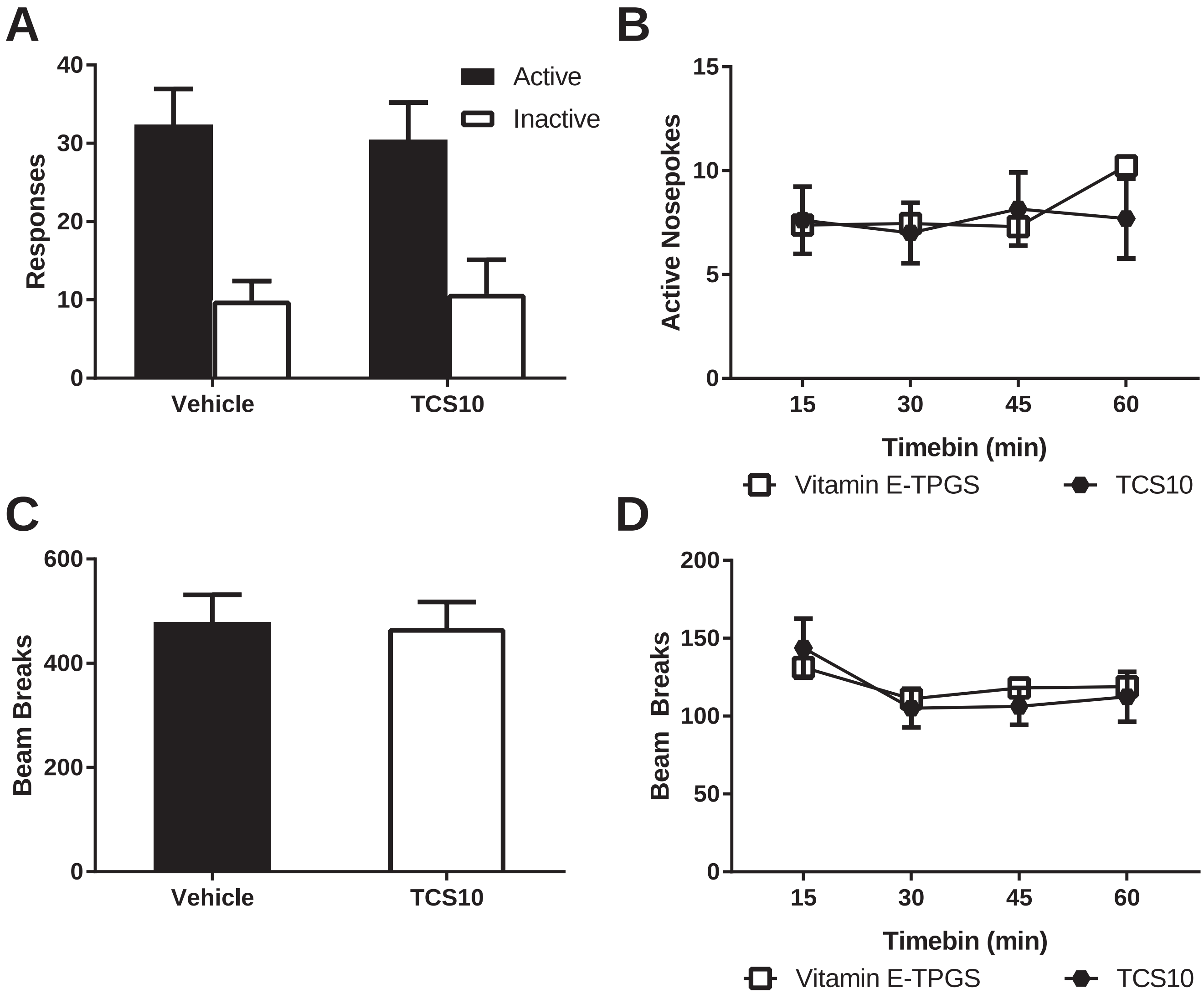

S1 Fig 1. Replication of self-administration test using 20% Vitamin E-TPGS/0.9% saline vehicle. After self-administration testing, the highest dose was replicated using a 20% Vitamin E-TPGS/0.9% saline vehicle. (A) No difference was observed on either the total number of active and inactive nosepokes. (B) No alteration to the timecourse of responding was observed. (C) No difference in locomotor activity was observed during the operant session. (D) The timecourse of locomotor activity was also not altered.
